# Dynamical Network Models From EEG and MEG for Epilepsy Surgery—A Quantitative Approach

**DOI:** 10.3389/fneur.2022.837893

**Published:** 2022-03-29

**Authors:** Miao Cao, Simon J. Vogrin, Andre D. H. Peterson, William Woods, Mark J. Cook, Chris Plummer

**Affiliations:** ^1^Center for MRI Research, Peking University, Beijing, China; ^2^Department of Medicine, The University of Melbourne, Melbourne, VIC, Australia; ^3^Centre for Clinical Neurosciences and Neurological Research, St Vincent's Hospital Melbourne, Melbourne, VIC, Australia; ^4^School of Health Sciences, Swinburne University of Technology, Melbourne, VIC, Australia

**Keywords:** dynamical network models, non-invasive, EEG, MEG, epilepsy, epilepsy surgery

## Abstract

There is an urgent need for more informative quantitative techniques that non-invasively and objectively assess strategies for epilepsy surgery. Invasive intracranial electroencephalography (iEEG) remains the clinical gold standard to investigate the nature of the epileptogenic zone (EZ) before surgical resection. However, there are major limitations of iEEG, such as the limited spatial sampling and the degree of subjectivity inherent in the analysis and clinical interpretation of iEEG data. Recent advances in network analysis and dynamical network modeling provide a novel aspect toward a more objective assessment of the EZ. The advantage of such approaches is that they are data-driven and require less or no human input. Multiple studies have demonstrated success using these approaches when applied to iEEG data in characterizing the EZ and predicting surgical outcomes. However, the limitations of iEEG recordings equally apply to these studies—limited spatial sampling and the implicit assumption that iEEG electrodes, whether strip, grid, depth or stereo EEG (sEEG) arrays, are placed in the correct location. Therefore, it is of interest to clinicians and scientists to see whether the same analysis and modeling techniques can be applied to whole-brain, non-invasive neuroimaging data (from MRI-based techniques) and neurophysiological data (from MEG and scalp EEG recordings), thus removing the limitation of spatial sampling, while safely and objectively characterizing the EZ. This review aims to summarize current state of the art non-invasive methods that inform epilepsy surgery using network analysis and dynamical network models. We also present perspectives on future directions and clinical applications of these promising approaches.

## 1. Introduction

Epilepsy is a debilitating neurological disorder that affects 1–2% of the population worldwide (1). About two thirds of epilepsy patients may have their seizures controlled using anti-epileptic drugs (AEDs), while at least one third of patients do not adequately respond to medications (2, 3). More crucially, this ratio of pharmaco-refractory patients has not changed with the introduction of new first-line AEDs each year (4). For those pharmaco-refractory patients, surgical intervention (with the removal of brain tissue driving ictogenesis) can serve as a viable option for the treatment of drug-refractory epilepsy (5).

The success rate of epilepsy surgery is between 30 and 70% (6, 7). A recent multi-center study suggests the success rate of epilepsy surgery is about 50% (8). While the role of epilepsy surgery is well-established, the estimated ratio of operated to potentially eligible patients is only 1:25–50 (9). Accurate localization of the epileptogenic zone (EZ)—the minimum brain area to be removed to render a patient seizure free—is the ultimate goal in the pre-surgical evaluation of these patients (5, 10). Invasive intracranial monitoring (with direct recordings of local field potentials generated by pathological brain tissue) is still the gold standard to delineate the EZ presurgically (1, 5, 6). However, it is not a true gold standard because intracranial recordings have multiple key limitations (11). These include high cost, significant patient morbidity, and the element of subjectivity involved in the identification of the iEEG-defined seizure onset zone (SOZ) (8, 11). The analysis of ictal iEEG is typically restricted to visual inspection; however, a more objective approach to the analysis of iEEG data is beginning to emerge in the clinical setting (12–14). For instance, a number of investigators have developed quantitative approaches (12–14) to the analysis of clinical EEG to reduce the degree of subjectivity involved in the clinical interpretation of these complex datasets. Of the various forms of iEEG (classical sEEG, isolated depth electrodes, intraoperative monitoring, subdural grids, and strips), it is sEEG (with its more extensive sampling capacity) that has fostered a deeper understanding of the network nature of the EZ, challenging the clinical view that the EZ is a discrete unifocal zone.

Network analysis and network models have assumed important roles in the present-day imaging of brain networks and their functions (15–17). As a fast-evolving research area, the recent advances in network analysis and network models enable the study of both normal and pathological brain dynamics by taking into account high-dimensional information obtained using neurophysiological and neuroimaging approaches (18–20). Aided by techniques from neuroscience and neuroimaging, a large number of studies using network analysis and network models have shed new light on our understanding of the enormous complexity of the epileptic brain (21).

Dynamical network models provide great capacity to probe the mechanisms underlying complex neural dynamics (15, 17, 22, 23). Inspired by pioneering studies of excitatory and inhibitory neurons as well as the alpha rhythm of the thalamus (24–26), investigators have developed dynamical models of neural mass and neural mass networks, which connect an ensemble of neural mass models into macroscopic neural systems (27, 28). Employing dynamical network models, multiple attempts have been made to understand the mechanisms underlying normal and pathological neural dynamics (29–34). Dynamical network models have also been applied to neurophysiological data recorded from the human brain to develop specific hypotheses toward clinical application (20, 29, 31, 35, 36). In this review, recent advances and notable developments in the field will be examined in the context of epilepsy surgery.

## 2. A Generic Workflow

**Figure 1:**
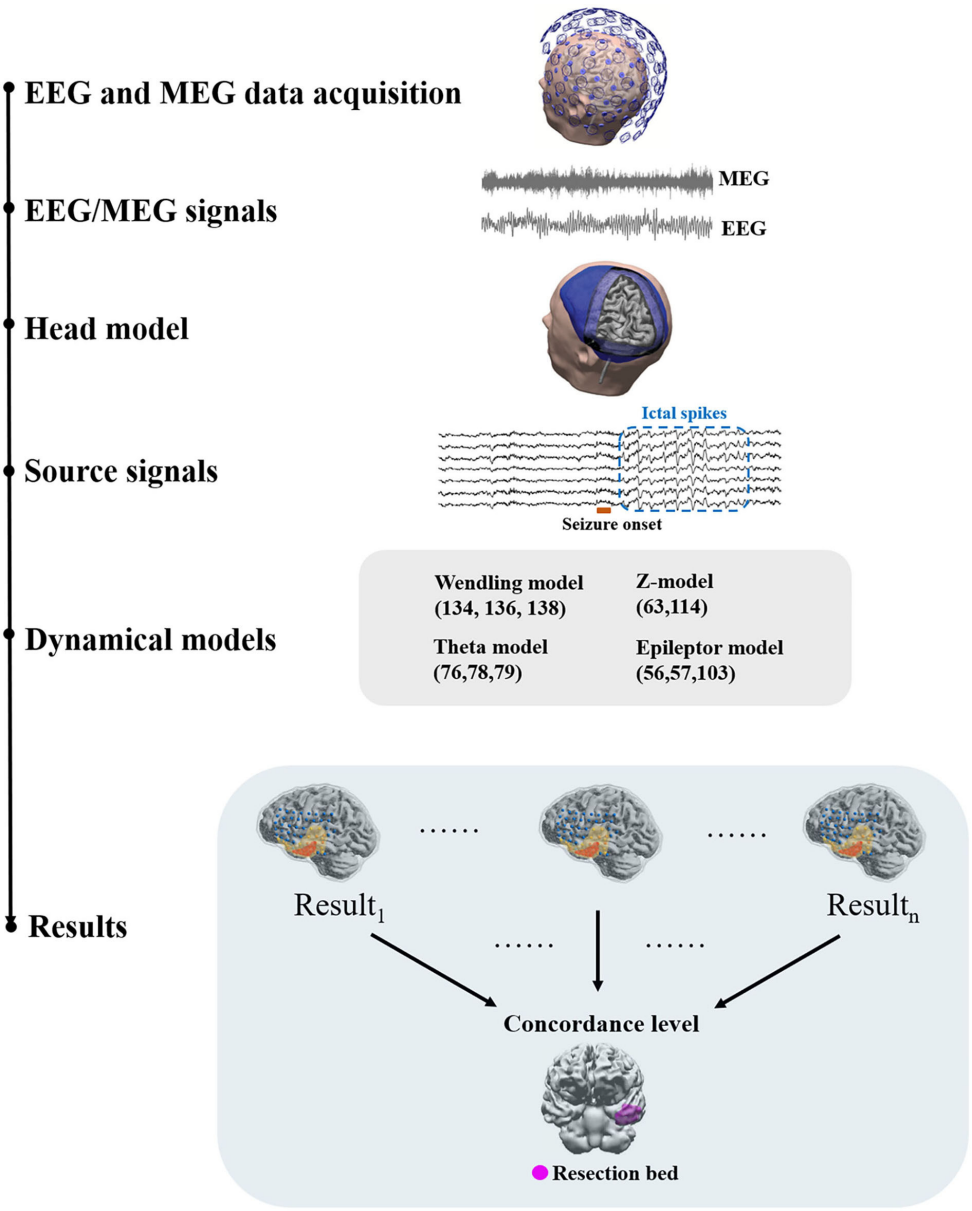
A generic workflow. EEG and MEG signals are acquired for presurgical evaluation. Preprocessing of EEG and MEG signals is often required before source modeling to remove artifacts. The head model and the co-registered source space are then prepared using individual structural MRI data to generate a forward solution. Inverse solutions can be then generated using forward solutions and EEG/MEG signals. Using inverse solutions, source activity can be localized and reconstructed. Next, functional networks can be constructed using source signals and dynamical network models can be applied to identify brain areas that are responsible for ictal or interictal discharges. Dynamical network models can be then clinically validated against surgical resection margins linked to histology and post-surgical outcome.

A generic workflow of applying network analysis and dynamical network models to EEG and MEG source signals is depicted in Figure 1. EEG and MEG signals acquired as part of the presurgical evaluation are first preprocessed *via* multiple steps before they are source modeled (37). After preprocessing, the head model and source space are constructed using the individual's MRI data. Forward and inverse solutions are then generated for source imaging. Source signals in defined source space can be then reconstructed. With reconstructed source signals, functional networks can be constructed using connectivity approaches.

Network modeling generally requires a connectivity analysis to obtain a network structure or topology as the basis of the modeling as the first step. This network structure may come from structural imaging data such as tractography or functional connectivity. When using functional connectivity to determine network structure, a series of time-evolving functional networks may be used (19) or a time-domain averaged functional network may be used. Some models also offer the capacity to use directional networks and hence effective connectivity and causal relationships may be integrated into the network structure (33, 38). Nodal level neural dynamics can then be embedded into network nodes. Multiple models of neural dynamics using different mathematical mechanisms can be employed in this step. Some models also offer flexibility by accommodating the use of different models to configure nodal neural dynamics. Network simulations can then be run with or without external inputs, such as perturbatory white noise. By introducing external noise, “stimulation,” or change in parameters, models can effect a transition from non-seizure states to seizure states (39–42).

**Figure 2:**
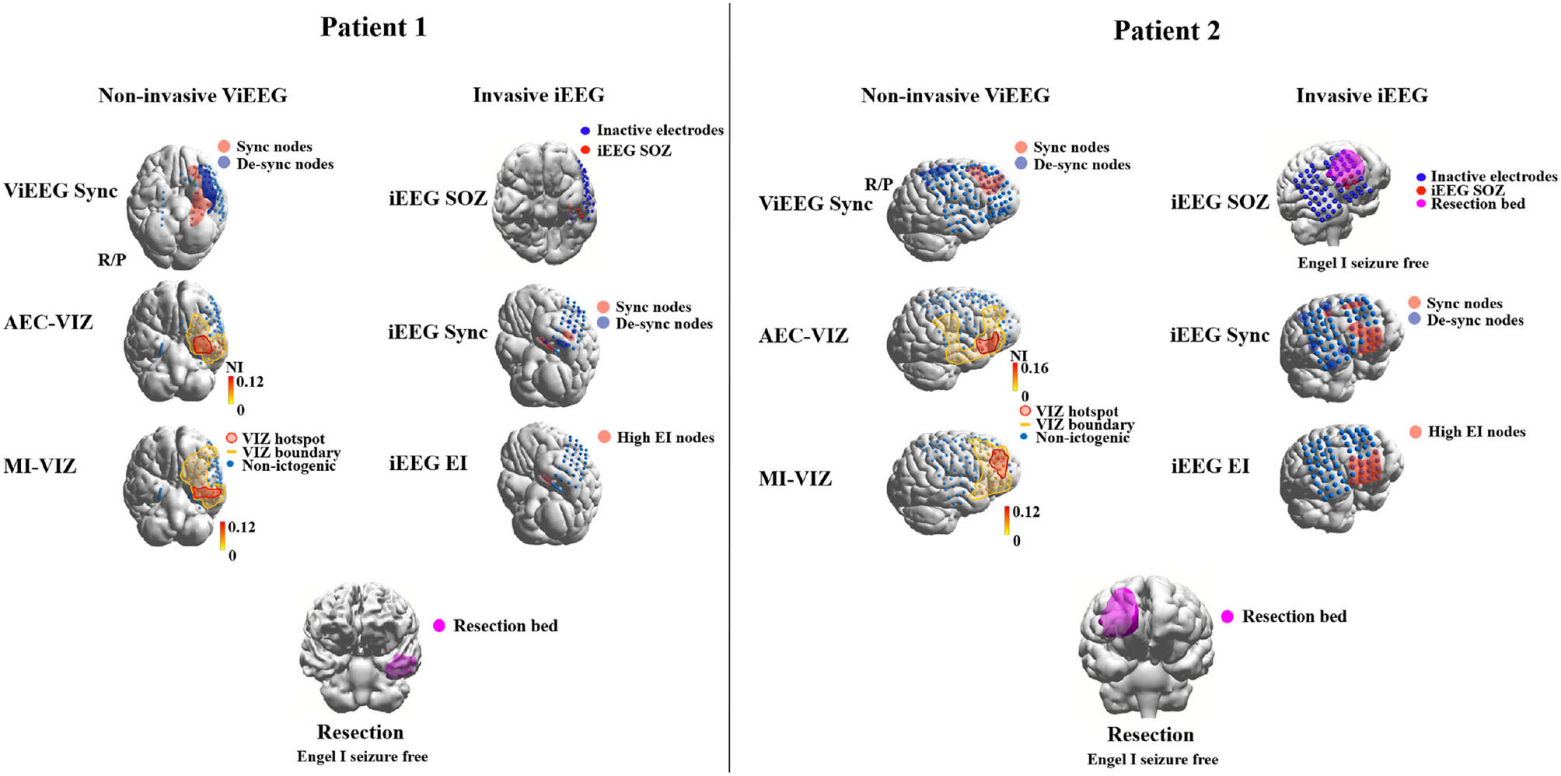
Examples of applying dynamical network models to non-invasive (MEG) and invasive (iEEG) data to identify brain areas that are responsible for ictogenesis. Three approaches are applied to MEG and iEEG data, respectively, to identify brain areas that are responsible for seizure generation (red highlight). These areas are then compared against the resection margin and surgical outcomes to validate the results of employed approaches. The Sync approach uses synchronizability and control centrality (19) to identify nodes that increase or decrease of the stability of the synchronous states of the network. AEC-VIZ and MI-VIZ represent the ictogenic zone identified using virtual iEEG signals reconstructed by ictal MEG and dynamical network models. Amplitude Envelope Correlation (AEC) and Mutual Information (MI) can be used to construct functional networks that are then fed to dynamical network models. Here, a Theta model is used to simulate ictal waveforms and a virtual resection technique to estimate the influence of each node on ictogenicity. The Epileptogenicity Index (EI) (43) estimates spectral and temporal features of ictal iEEG signals and provides a quantitative measure to identify epileptogenic areas. iEEG SOZ is the conventional clinical analysis of ictal iEEG signals to identify iEEG electrodes where seizures arise. For both patients, brain areas involved in epileptogenesis identified by noninvasive dynamical approaches are comparable to the areas identified by traditional invasive intracranial means. Both patients had an Engel 1 outcome—Patient 1 **(left)** had focal cortical dysplasia Type 1 and Patient 2 **(right)** had post-infectious cortical gliosis.

Each model generates a probability map that depicts the likelihood of brain areas being responsible for interictal or ictal source activity depending on the nature and the assumptions that a method or model employs. Such probability maps can then be used to assess the concordance level with resection bed. Using concordance levels and post-surgical outcomes, the performance of models and approaches can be tested. Two patient examples are given in Figure 2.

## 3. Functional and Structural Networks in Focal Epilepsy

With ongoing advances in neuroimaging techniques, high-resolution functional and structural neuroimaging data can be obtained from epilepsy patients for assessment, diagnosis, treatment, and research. Connectivity methods have commonly been used to construct functional and structural networks using neuroimaging data. This subsection discusses findings from studies using functional and structural connectivity and problems and limitations associated with connectivity analysis in epilepsy.

### 3.1. Structural Networks in Focal Epilepsy

When studying functional brain networks, an intuitive question to ask is how structural networks constrain functional networks. MRI techniques and structural connectivity have been introduced to address this. MRI techniques are widely used in clinical workup to localize pathological brain regions and understand epileptogenesis (1, 44). Diffusion MRI (dMRI) is a variant of standard MRI and one of the mainstream structural imaging techniques (45).

In a typical connectivity analysis, a standard MRI scan is required to capture an individual's neuroanatomical structure. Analytical software, such as Freesurfer (46), can be used to segregate the whole brain into subregions based on a standard brain atlas or customized boundaries (47, 48). With dMRI, software that tracks fiber density or integrity can be then used to detect, count and quantitatively characterize fibers that communicate between parcelated brain regions. This fiber density analysis results in a two-dimensional connectivity matrix, representing how strongly subregions are interconnected *via* white matter. This two-dimensional connectivity matrix may become a “fingerprint” of an individual's structural networks. Properties of the individualized connectivity matrix may characterize critical features of a pathological brain (49, 50). Early studies using dMRI and connectivity analysis suggest a change in structural connectivity in the epileptogenic zone and surrounding brain regions in focal epilepsy (44, 51, 52).

More specifically, in temporal lobe epilepsy (TLE) patients, structural alterations were reported in the epileptogenic zone in frontal and temporal lobes, but particularly at the temporal poles. These structural alterations revealed by tractography and connectivity analysis indicate distinct unilateral features and specific impacts on global structural connectivity (52). Despite variance introduced by individual differences and heterogeneous pathologies in group-level analysis, studies comparing TLE patients and healthy cohorts demonstrate extensive weakened temporo-parietal connections in TLE structural networks, which support the clinical observation of cognitive impairment in memory and speech (53, 54). Focke et al. (54) also demonstrate altered structural connectivity between para-hippocampal structures, providing a neuroanatomical basis for theoretical models of seizure propagation. In frontal lobe epilepsy, structural connectivity may remain intact in frontal regions, while nearby regions can be affected by interictal and ictal activity (55). Epilepsy involving mesial frontal areas preserves a robust connectivity in the supplementary motor area. Similar lower fiber intensity found in the superior longitudinal fasciculus, but not in the cingulum, suggests particular functional abnormalities for children with focal epilepsies (56). Diffusion imaging may also be used in animal models to study the extent of white matter impairment. A rat model of focal epilepsy has been studied using dMRI and shows widespread reductions in white matter density in extensive brain regions beyond the epileptic focus, indicating the impaired efficiency of functional networks (57). Animal models, however, are not the focus of this review.

Structural networks defined by structural connectivity are not a complete representation of a pathological brain. Due to limitations of current techniques, structural MRI can only capture a small proportion of network connections on the macroscopic level. Whether this limitation affects interpretation of current studies in focal epilepsy remains unclear (45). Computer simulations show structural alterations are not necessary to generate seizure-like activity and epileptic networks are also believed to be fast-evolving dynamical networks (58). Therefore, rather than characterizing interconnected brain regions, static structural connectivity is more likely to answer how functional networks can be constrained by their corresponding structural substrates. This is important to keep in mind when interpreting findings on functional networks in focal epilepsy.

### 3.2. Functional Networks in Focal Epilepsy

Previous studies using functional connectivity mainly focus on time-series analysis of interictal and ictal events and report on network structural alterations over time before, during and after a seizure. To gain insights into the fast-evolving functional networks of seizure activity, recording techniques with high sampling rates, such as EEG, iEEG, and MEG, are broadly employed for epilepsy research. A number of studies have demonstrated the capacity of functional network structures of fast-evolving seizures to reflect properties of the putative EZ (13, 18, 58–61).

Using sEEG recordings, Bartolomei et al. (14) are credited as the first study to apply network analysis to explore non-linear relationships between different brain regions in temporal lobe epilepsy patients. Bartolomei et al. (62) offers a comprehensive review of network analysis specific to sEEG in epilepsy surgery. Khambhati et al. (19) show functional connectivity changes rapidly over time before focal seizure onset but not as much as it does during the seizure. By clustering time windows of iEEG data based on functional connectivity commonalities, Khambhati et al. (63) also find higher levels of synchronization in brain states that are close to focal seizure termination as opposed to brain states at the beginning or the middle of the seizure. These findings indicate that the epileptic brain has different functional network structures underlying seizure generation vs. termination. Schindler et al. (64) also demonstrated the shift in functional network structure toward a normal network state with transition from the pre-ictal to the ictal state.

Studies have reported that the ictal network structure for generalized seizures was more regular than the corresponding interictal network structure, thus suggesting that seizure events with seemingly “random” functional connectivity may preserve common patterns (65–67). Distinct patterns of functional connectivity have also been reported around seizure onset. Kramer et al. (68) demonstrated the SOZ presents a dominant regular sub-network with densely connected nodes. As a seizure progresses, the sub-network becomes divided into smaller random networks and hence the authors argue that these network features during the seizure progression may reflect decreased susceptibility of the network to become synchronized (68).

Interictal brain networks have also been examined in functional network studies. Resting-state EEG and MEG recordings in focal epilepsy patients show an increase in functional connectivity, which could reflect increased cortical excitability predisposing to epileptic seizures (69, 70). These authors also identified a decrease in network efficiency compared to control networks, perhaps indicating brain network disruption associated with interictal activity. Others employing network analysis of interictal data report conflicting results. Bartolomei et al. (71) presented decreased clustering coefficients and path lengths, while Horstmann et al. (72) show an increase in the same metrics. These inconsistencies could be due to differences in patient selection and methodologies. Current techniques representing functional networks may well need further refinement to characterize a pathological brain, particularly a brain predisposed to seizures.

Functional networks have also been studied using fMRI in generalized and focal epilepsies. In temporal lobe epilepsy, a general decrease in functional connectivity has been reported in the ipsilateral hemisphere and subcortical structures (73, 74). Another study reports that besides a general decrease in global functional networks, there is a relative increase in functional connectivity within the affected temporal lobe (75). In generalized epilepsy, a decreased intra-hemispheric connectivity and an increased inter-hemispheric connectivity are reported (76). Although associations between hemodynamic signals and electromagnetic signals require more investigation, these fMRI findings provide a different perspective on network behavior based on interictal data.

### 3.3. Relationship Between Structural and Functional Connectivity and Limitations of Connectivity Analysis

To date, a well-defined relationship between functional and structural connectivity is still missing in the literature for several reasons. First, functional connectivity in meso-scale brain networks still lacks sufficiently accurate neurophysiological and neuroanatomical substrates to interpret findings; to begin with, the coupling of structural to functional networks is not straightforward as findings from structural connectivity may not directly translate to neural dynamics governing interictal and ictal states. Second, structural connectivity is not always static and, as revealed by work in neuroplasticity, can change over longer time scales. Therefore, studies with different follow-up protocols are not always comparable. Third, individual differences make findings difficult to generalize statistically, especially when dealing with pathological substrates. For example, pathologies residing in different cortical regions may result in different functional and structural network structures complicating group analysis and potentially introducing errors to epileptic network modeling at the level of the individual. Such limitations of current connectivity analysis make it difficult to clearly define the extent by which structural connectivity constrains functional connectivity. In the context of epilepsy, multiple factors potentially influence connectivity analysis findings. For example, the effect of anti-epileptic drugs (AEDs) on functional and structural connectivity is unclear (59). Heterogeneity of epilepsy patients is also non-trivial. Different lesion types and locations might exert different effects on functional and structural connectivity properties (77). Normally in epilepsy studies, patients with the same pathology and similar locations are grouped and studied together. Patients with the same pathology and similar locations may have very different ictal or interictal electrographic activity, while patients with different pathologies may demonstrate similar electrographic features (59). These factors need to be considered when validating network models in cohorts obeying conventional patient selection.

Contradictory results from different imaging modalities also influence how findings should be interpreted. EEG and MEG studies usually show global increases in functional connectivity compared to healthy controls, while fMRI studies show a general decrease. This might reflect fundamental differences between hemodynamic coupling and electrophysiological dynamics in epilepsy, not least in their respective temporal and spatial resolutions (78). Future studies that assess the relationship between neurophysiologic and hemodynamic connectivity are needed, possibly through simultaneous multi-modal neuroimaging studies (16, 59, 79, 80).

## 4. Network Analysis of Functional Brain Networks

Networks are an abstract mathematical construction that aim to represent the interaction of complex real-world systems. This concept has been introduced to many disciplines including physics, biology, ecology and neuroscience, to describe the mathematical behavior of complex systems. In neuroscience, networks are generally derived from functional and structural connectivity pathways, where “nodes” stand for different brain regions and “links” represent anatomical paths between brain regions or statistical correlations between neural activity (81). Network analysis using graph theoretical metrics, for example, has offered insights into how different brain regions are structurally connected and how different brain regions interact with each other spatio-temporally (49).

Over the last 5 years, network analysis has become a hot topic in clinical neuroscience research, as a pathological brain shows distinct features in structural and functional networks against a healthy brain (66, 67, 82). These brain network features can be used as biomarkers for clinical application. As epilepsy is becoming more recognized as a brain network disorder, network analysis allows us to study epilepsy and epileptic seizures from a novel perspective (18, 83, 84). The next section discusses how to define networks using connectivity methods and extract network features using graph-theoretical metrics. It also discusses findings and their interpretation from network analysis, and potential biomarkers that can be used for clinical applications.

### 4.1. Nodes and Edges

A network is composed of nodes and edges that link nodes. In functional brain networks, nodes stand for different brain areas and edges stand for functional dependence between regional activities (85). The way nodes and edges are defined often depends on the imaging modality that is used. For example, with fMRI, we can use a voxel, or several neighboring voxels as a node (86); independent component analysis (ICA) can also be used to aggregate voxels into nodes (87). Time-series of nodes that have the same independent component can be aggregated into a node. For sensor-based modalities, such as EEG and MEG, the preference is to directly use sensors as nodes or assign nodes in reconstructed source space (88). Brain parcelations of structural MRI also provides a sophisticated means of assigning brain areas to nodes, although this requires a-priori knowledge of individual brain structures and a standard brain atlas (89).

Edges are typically estimated by quantifying statistical dependency of neural activity between two regions (90). However, edges are not necessarily equal to connectivity matrices, as network edges can be binary (edge is either zero and not connected, or one and connected) or weighted (when normally a graph filter is applied to extract important edges). The reason to apply a graph filter is that functional connectivity can be affected by noise and other measures and graph filtering can remove such connections (91).

There are multiple ways to apply graph filters to brain networks. Setting a threshold to connectivity matrices can extract dominant connections. However, one of the problems with setting a hard threshold to a matrix is that edge weights can significantly increase or decrease depending on the brain state. Therefore, a constant hard threshold for different time windows may bias global network structures. Proportional thresholding can help with time window problems as it iteratively extracts top-ranked connections. However, a common problem of network thresholding is that without defining connections of interest, dominant connections across a certain time window could be irrelevant to analysis or might have even been generated by artifacts (92). In an effort to address such issues, Langer et al. (92) proposed the use of sophisticated statistics in their study, but given the enormous complexity of neural activity, it is difficult to select neural activities that are relevant for study by examining whether or not they are statistically correlated.

### 4.2. Graph Theoretical Metrics

With established functional networks, graph-theoretical metrics can be applied to study network properties. A number of graph-theoretical metrics have been developed to measure different network topological features and each of them has specific assumptions and requirements of the network (81). In general, graph-theoretical metrics extract four categories of network features: integration, segregation, motif, and centrality (93). For example, clustering coefficients and community detection metrics quantify how densely subgroups are connected in a network. Shortest path metrics, such as global efficiency and characteristic path length, estimate levels of network integration. Betweenness centrality and closeness centrality detect important hubs that bridge multiple sub-groups. Different metrics, by their definition, extract different network properties, as shown in Table 1.

**Table 1:** Commonly used graph-theoretical metrics and their scales, features, and requirements (81, 93).

**Scale**	**Metric**	**Features**				**Requirements/Connected**
		**Category**	**Weighted**	**Directed**	**Negative**	
Whole brain network	Characteristic path length	Integration	Yes	Yes	No	Yes
	Global efficiency	Integration	Yes	Yes	No	No
	Clustering coefficient	Segregation	Yes	Yes	No	No
	Local efficiency	Segregation	Yes	Yes	No	No
	Modularity	Segregation	Yes	Yes	Yes	No
Sub-networks	Motifs	Motif	Yes	Yes	No	No
	Transitivity	Segregation	Yes	Yes	No	No
	Edge betweenness	Segregation	Yes	Yes	No	No
Nodes	Degree	Basic metric	Yes	Yes	No	N/A
	Number of triangles around a node	Basic metric for segregation	Yes	Yes	No	N/A
	Shortest path length	Basic metric for segregation	Yes	Yes	No	N/A
	Closeness centrality	Centrality	Yes	Yes	No	No
	Betweenness centrality	Centrality	Yes	Yes	No	No

Selecting appropriate graph-theoretical metrics in studies is non-trivial. This metric selection normally depends on the research question, assumptions, and hypothesis (78). Several questions may be asked when choosing metrics, such as does the study focus on whole brain networks or sub-region networks? Is the study assuming its networks are fully connected or operating as isolated nodes or sub-groups? Does the study look at important nodes in networks? Specific hypotheses may lead studies to mainly look at a subset of nodes and edges, which may require tailored metrics to extract features of interest. Metric selection should also consider what imaging modality functional networks are derived from. Just as different imaging modalities have different spatio-temporal resolutions and reflect neural dynamics at different spatio-temporal scales, functional networks have different features and properties (94). Graph-theoretical metrics applied to these networks should take the inherent assumptions of specific network properties into account.

Thorough statistical tests of network models are critical. There are two ways of testing network models: (1) compare against numerically simulated reference models and (2) compare with models derived from other conditions, such as task vs. resting-state or healthy vs. pathologic (93). A statistically “null model” is often used as a reference model to test whether the phenomena that a model observes is random (95). However, a null model is not always statistically random. A null model is often assigned properties that the derived model shares. For example, a null model normally has the same node degree distribution and similar modular structure. Although network link weights of a null model usually remain random, they still follow distributions of the derived model (96).

### 4.3. Interpretation and Biomarkers

A question that is often raised when results are obtained from network analysis is how to interpret findings. Unfortunately, this question is not easy to answer. As discussed in previous sections, connectivity methods and graph-theoretical metrics reduce the dimensions of neuroimaging data but also increase levels of abstraction (97). Although new information can be obtained with higher levels of abstraction, we also lose the ability to directly interpret results and to understand neurophysiological substrates (98). Specifically, a small change in original neural signals will propagate through levels of abstraction, along with added complexity. In other words, any change at a high level of abstraction may not have a one-to-one mapping to original signals. Current studies use variable-control strategies to rule out factors that do not affect final results (99). However, this strategy may not be available when using complex approaches, such as network analysis. Interpreting results has remained a challenge in this area and current studies are generally conservative and cautious with interpretation.

Although interpreting findings from complex network analysis remains challenging, these findings can still be used as potential biomarkers for clinical applications. For example, functional and structural coupling and decoupling have been found to be complex and mechanisms remain unknown (100). However, distinct patterns of decoupled functional and structural network structures may reflect long-term impairment in idiopathic generalized epilepsy patients and may be used as a biomarker to detect subtle brain abnormalities (100). Zweiphenning et al. (101) found high-frequency functional networks have distinct biomarkers that statistically predict the location of the seizure onset zone using interictal iEEG data. These biomarkers are useful for patients who do not have frequent clinical or sub-clinical seizures on iEEG monitoring. Studies using network modeling and network analysis have also discovered biomarkers with the potential to predict outcomes of epilepsy surgery (19, 29, 32, 102). These biomarkers may prove to be useful for presurgical evaluation if findings can be validated clinically through prospective studies and clinical trials.

### 4.4. Volume Conduction and Source Connectivity

The biophysical nature of volume conduction from neural sources to recorded signals can introduce field spread or smearing in connectivity calculations, whereby instantaneously correlated signals are reconstructed in localized brain areas and spurious connections are identified by conventional connectivity analysis. The early work in the biophysics of brain volume conductor modeling for electrophysiological signals has discussed this issue and is summarized in the review by Vorwerk et al. (103).

Unfortunately, this issue is not alleviated when simpler forward solutions are applied to MEG source reconstruction. Volume conduction also raises the concern as to whether or not non-invasive source analysis can achieve the spatial accuracy of invasive intracranial approaches. This is because volume conduction smears the electrical potential field (as well as the magnetic field) generated by a current dipole in the brain, particularly when the smeared field is observed from far afield. Fortunately, volume conduction only “mixes” neural activity in a linear fashion with zero delay in phase synchrony. This opens the door to find ways to limit volume-conduction related spurious connections interfering with connectivity calculations. By understanding the principle of volume conduction, various techniques have been developed over the last two decades to remove instantaneous correlation and phase synchrony between a pair of signals (65, 104–108). Unfortunately, a recent study that assesses these techniques demonstrates that none guarantee full identification and removal of spurious connections (109). Some approaches perform better than others in certain simulated paradigms but these may also turn out to be too conservative to remove real connections (110). While volume conduction can complicate the use of brain network approaches for the study of neural mechanisms, some argue that volume conduction is not a major concern when a biomarker of a certain phenomenon is the goal.

### 4.5. Studies Using Network Analysis for Epilepsy Surgery

Early work by Kramer at al. (111) looked at pre-seizure, seizure and post-seizure functional networks in four patients and uncovered localized brain structures that appear to facilitate seizure generation. This finding suggested that network analysis can assist identification of pathological brain areas and potentially target these areas for surgical treatment (111). Later, Wilke et al. (112) used directional networks and graph theoretical metrics to investigate interictal and ictal iEEG networks. More recently, a new technique, virtual cortical resection, has been developed using functional networks and validated against clinical iEEG data (19, 63, 94, 113). By analysing functional connectivity patterns of ictal iEEG data, Khambati et al. (63) developed a framework that statistically describes network dynamics in seizure generation, propagation, and termination. The topographic and geometrical changes captured by their model suggest strengthened synchronous connectivity near foci may help seizure termination. This finding suggests that modulating certain circuits near pathologic foci may disrupt seizure propagation or control seizure generation. Khambhati et al. (19) later extended the network model by analysing focal seizures with and without secondary generalization. The authors hypothesized that focal seizures with secondary generalization are more likely to synchronize in the pre-seizure state and there is a regulatory network mechanism that controls whether a focal seizure generalizes secondarily. A measure, synchronizability, which has been used in stability analysis of complex systems (114), was used to quantify stability and heterogeneity of time-varying functional networks in the model. And a novel metric, control centrality, was proposed to quantitatively estimate how the synchronizability of a network changes when a node is virtually removed from the network (virtual cortical resection). Counter-intuitively, brain regions that regulate seizure dynamics and control secondary generalization were often found to sit outside the SOZ. The implication here is that surgical resection of the SOZ alone does not necessarily lead to long-term seizure freedom. Their novel approach also provides a framework to develop techniques that can computationally simulate epilepsy surgery in order to provide an optimal surgical strategy. Kini et al. (113) further extend the framework using ictal events from iEEG and provide a statistical bio-marker that supports the idea that synchronizing nodes in the network should be removed in surgery, pending overlap with eloquent cortex.

A study by Jiang et al. (115) independently revealed similar “push-pull” dynamics that regulate secondary generalization of focal seizures. Differing from the specific gamma band of Khambhati et al. (19), Jiang et al.'s (115) push-pull dynamics comes from within- and across- frequency oscillations. Sohrabpour et al. (20) applied network analysis to the EEG source space to provide a non-invasively derived prediction of the EZ.

Other studies (112, 116) use directional networks to identify a subset of brain areas for potential surgical removal. Hassan et al. (117) and Juarez-Martinez et al. (118) extend network approaches to EEG and MEG source space with relatively small numbers of patients compared to Sohrabpour et al. (20). These studies provide further insights into how network analysis can be translated from invasively recorded data to non-invasively recorded and ideally whole-brain data. Other network analysis studies using pre- and post-operative EEG, MEG, and fMRI data also found significant changes in functional connectivity patterns that were predictive of surgical outcomes (13, 18, 119, 120). A summary of studies using network analysis is given in Table 2 and a comparison of network analysis and network modeling approaches by modality and source-sensor space is given in Table 3.

**Table 2:** A summary of network analysis studies for epilepsy surgery.

**References**	**Functional network**	**Patient number**	**Clinical data**	**Pathology**	**Findings**	**Comments**
Bartolomei et al. (14)	Undirectional& directional	18	Ictal SEEG	Various pathologies in temporal lobes	Confirmation of network phenomena during temporal lobe epilepsy seizures	The first study that analyzed the network phenomena in focal epilepsy
Jiang et al. (115)	Directional	24	Ictal iEEG	Various pathologies and locations	Secondary generalization of focal seizures is regulated by cross frequency push-pull dynamics	Second publication in literature on push-pull mechanisms of focal seizure
Sohrapour et al. (20)	Directional	36	Interictal & ictal iEEG + numerical simulations	Various pathologies and locations		
Khambhati et al. (19)	Undirectional	10	Peri-ictal iEEG	Various pathologies and locations	Identify a push-pull mechanism that regulates focal seizure secondary generalization	First paper reported such finding
Kini et al. (113)	Undirectional	28	Ictal iEEG	Various pathologies and locations	Synchronizing nodes should be considered to remove in surgical planning	Subsequent work of Khambhati et al. 2016 (19)
Lin et al. (116)	Undirectional	13	Ictal iEEG	Not available		
Wilke et al. (112)	Directional + graph theory	25	Ictal and interictal iEEG	Various pathologies and locations		
Kramer et al. (111)	Undirectional	4	Ictal iEEG	Various pathologies and locations	Localized brain areas that facilitate seizures and potential target for surgical removal	Early work analysing functional networks of ictal events using iEEG
Juarez-Marineza et al. (118)	Undirectional + source imaging	9	Ictal sEEG + interictal MEG	Various pathologies and locations	Reproduce seizure onset zone non-invasively and potentially identify biomarker for EZ	First MEG non-invasive source space analysis
Hassan et al. (117)	Undirectional + source imaging	1	Ictal sEEG + ictal EEG	Not available	Identify epileptic focus that also matches findings from sEEG recordings	

**Table 3:** A comparison matrix demonstrates current state of each direction of network analysis and network models using different imaging modalities.

**Network analysis/Models**		**Network analysis**	**Network mode**
MEG	Source	Main field (diagnosis, prognosis, surgical strategy), but no comparison against source localization	No
	Sensor	Sensor-level analysis is more significantly affected by volume conduction and field spread than source space	No
Scalp EEG	Source	Main field (diagnosis, prognosis, surgical strategy), but no comparison against source localization (121) is the first study.	One study from Lopes et al. (122)
	Sensor	Main field (diagnosis, prognosis, seizure prediction)	Main field (diagnosis, prognosis, seizure prediction)
iEEG	Source	No	No
	Sensor	Main field (diagnosis, prognosis, surgical strategy)	Main field (diagnosis, prognosis, surgical strategy)

Despite the growing number of studies using network analysis for epilepsy surgical localization, prospective clinical studies are lacking. The numbers of patients included in studies has increased from one patient (117) to 36 patients (20). The retrospective nature and modest number of patients combine to limit the applicability and generalizability of network analysis approaches to clinical work-up for epilepsy surgery.

## 5. Network Models for Epilepsy Surgery

Dynamical network modeling is a branch of network science employing mathematical and computational techniques to depict, analyse and understand the dynamical behavior of the network i.e., how a specific network structure impacts on the system behavior, particularly state transitions and bifurcations, through a set of evolution equations that yields quantitatively accurate depiction and prediction (123). Such techniques enable the properties of patient-specific functional network structures to be interrogated and the ensuing dynamics to be explained and predicted. In the case of diseased brain networks such as epilepsy, the evaluation and prediction of pathological state transitions such as seizures is invaluable in a clinical context such as epilepsy surgery. As opposed to network analysis, network models use established network structures as a basis and embed dynamical mathematical models to network nodes coupled by edge weights to simulate overall network dynamics. The process uses static functional networks derived from time-series data to a dynamical mathematical system that changes over time such that various states of brain networks can be numerically simulated for analysis. Here we present established network models for epilepsy surgery and include studies that have applied these models to empirical data.

### 5.1. Network Models

Four main network modeling techniques have been applied to epilepsy surgery: “Virtual Epileptic Patient” using the “Epileptor” model from Jirsa et al. (36), “Virtual Cortical Resection” model using network synchronizability and control centrality from Khambhati et al. (19), a computational model using network excitability from Goodfellow et al. (29) and another computational model similarly using network excitability from Sinha et al. (31).

The Virtual Epileptic Patient (VEP) model is a hybrid model using a phenomenologically derived neural field model, the Epileptor model (124). Each network node is defined in combination with structural networks and hypotheses derived from MRI lesions and other clinical information. This model uses the theory of fast-slow non-linear dynamics to characterize the bifurcations for seizure onset and offset. The VEP model demonstrates the prediction of ictal spatial patterns and confirmation of presurgical hypotheses (30, 124, 125), which may benefit presurgical evaluation and planning of invasive intracranial monitoring. It models epileptiform discharges in computational simulations and identifies the similar bifurcation mechanisms that produce epileptiform discharges using real data. The Epileptor model has demonstrated a capacity to predict seizure propagation using ictal sEEG data (124, 125).

Later work (21, 30, 36, 126) proposed an individualized whole-brain model that incorporates functional and structural network models. The Epileptor signifies an advance in mathematical modeling of epileptic seizures not only because the model provides a form of taxonomy of seizure activity using nonlinear coupled oscillators, but it also provides a mathematical etiology of seizure dynamics. Another advantage of this Virtual Epileptic Patient (VEP) is that, by combining the modeling of neural dynamics with the modeling of structural networks, the approach provides explanatory and predictive capacity in a clinical setting. Using sEEG combined with structural imaging modalities, this integrated approach virtually reproduces the seizure spread over the network that predicts the EZ (36). It is worth noting though that the VEP model requires sophisticated iEEG and neuroimaging workup and demands much of computing resources.

Although neuroimaging modalities, including DTI and fMRI, have been routinely used by some centers in presurgical epilepsy workup, scanner availability and scanning time are still limited in many surgical centers, especially those in developing countries. Despite the limitations of the VEP model, the findings encourage the use of the VEP model in a multi-center clinical trial. Such an integrated approach has the potential to be extended to the study of normal brain networks and to other neurological diseases.

The virtual cortical resection model provides specific insights into seizure evolution, particularly seizure initiation, and termination (19). Unlike the Virtual Epileptic Patient (36), the virtual cortical resection model only uses data from invasive intracranial recordings. By converting intracranial signals into fast evolving functional networks over time, two network metrics from network control theory (synchronizability and control centrality) are used to explore the contribution a node makes to the network dynamics. The virtual resection technique employed Master Stability Function (MSF) to estimate stability of synchronization (i.e., synchronizability) by looking at eigenspectra over time. However, MSF treats each node in the network as identical and synchronized and hence, is less concerned with individual dynamics (127). By correlating the mathematical change in functional network structure to clinical resection margins and surgical outcomes, the model suggests network nodes with high control centrality are likely to be included in the resection when a patient achieves a favorable outcome. The synchronizability values of functional networks using data before seizure onset successfully predict whether a focal seizure secondarily generalizes. This model provides important insights into this field. It offers an objective approach for surgery and carries the potential to optimize the surgical strategy.

The computational model from Goodfellow et al. (29) uses the Wendling Model (33, 128) to describe nodal level neural dynamics from functional connectivity analysis of ictal iEEG signals. While each node has the same dynamics characteristics, the network topology determines how the network transitions from the non-seizure state to the seizure state. The model is calibrated to assume that 50% of the nodes in the network transition into a seizure state with the whole network spending 50% of its time in a seizure state (29, 35). The total amount of time the network spends in the seizure state may increase, decrease, or remain the same when the network topology is changed with the removal of a given node. The assumption of this model is that virtually removed nodes that shorten seizure state time should be removed to reduce the risk of ictogenesis. A series of studies based on the theta model (35), which is a simplified version of the Wendling model, showed a correlation between model prediction and surgical outcome. By doing so, the model offers an opportunity to optimize surgical strategy for cases with unfavorable surgical outcomes. Another computational model from Sinha et al. (31) uses a similar mathematical framework (23, 129) to predict surgical outcomes and alternative surgical strategies.

### 5.2. Studies Using Network Models for Epilepsy Surgery

The work from Goodfellow et al. (29) and Jirsa et al. (36) are the early attempts to apply network models to intracranial data obtained for epilepsy surgery. These fundamental contributions motivated by earlier theoretical work (23, 124, 129–131) led to a series of publications aiming to more objectively and accurately predict the EZ. Goodfellow et al. (29) employed a full Wendling model to simulate excitability at the nodal level and predict surgical outcomes based on degree of overlap between model-predicted ictogenic nodes and resection margins. The study suggested that at least one node of high ictogenicity should be included in the surgical resection to achieve a more favorable surgical outcome. To better understand the relationship between SOZ and EZ, another measure, Seizure Likelihood was developed together with an earlier measure, Node Ictogenicity (NI) (29) to systematically compare the SOZ with the EZ. It was found that the SOZ may not be the best predictor of the EZ when there is significant heterogeneity in network topology and node excitability (132). This is perhaps in line with the clinical observation that SOZ-based resections do not always provide optimal outcomes (5). A later study on the same dataset reveals that a so-called “rich-club” organization (133) (a structure with multiple hub nodes that densely interconnect sub-networks) can be found in epilepsy surgical candidates and that disruption of rich-club modules might optimize surgical outcomes (35). This finding is also predicted by simulations using the same theoretical model that is simpler than the Wendling model. The most recent work by Lopes et al. (122) has extended their network model to non-invasive EEG source space. Using a simplified Wendling model and minimum-norm estimation, EEG source signals are modeled in a similar fashion to iEEG signals. Their results suggest that the network model predicts the lateralization of epileptogenic sources with modest spatial resolution. This work represents an important step in the effort to more objectively characterize the EZ non-invasively using source space signals and network models.

By extending the work of Jansen et al. (134) to also include a slow inhibitory population, Wendling et al. (129) model seizure onset by mathematically simulating the fast and slow oscillations of both excitatory and inhibitory neuronal populations. This model was used by Terry et al. (23) to inversely fit intracranial EEG data. Bettus et al. (69) and Wendling et al. (135) also applied the model to both intracranial EEG and scalp EEG. Wendling et al. (136) then extend the network model to understand seizure generation and propagation networks. More recent work has looked at the effects of disrupting network nodes that regulate seizure propagation (19, 113, 137) with results that challenge the traditional approach of SOZ resection as best practice for epilepsy surgery (19, 29, 94, 113, 132).

A multi-level computational model has lately been proposed to better replicate observed signals from experimental data for improved prediction of ictogenesis. This network model has been extended to EEG source space with promising results that reflect a good match between the interictal EEG source network and the interictal sEEG network (138). The study also found that the multi-level network model performs better in the localization of multi-focal epilepsy.

### 5.3. Summary of Network Models for Epilepsy Surgery

It is difficult to compare different studies using network models to predict the EZ owing to differences in the initial modeling assumptions and variation in patient cohorts, iEEG approaches, pathologies, and post-operative follow-up. The dominance of small studies and single case reports also limits the translatability of these approaches to the clinical setting. As presented in Table 4, there is accumulating evidence that network models can (a) predict the EZ using invasive neurophysiological data and non-invasive EEG data, (b) help unravel mechanisms of ictal and interictal discharge generation and propagation, and (c) allow the study of brain networks to be conducted in a patient-specific fashion. Long-term prospective studies are now needed, particularly with network modeling approaches based on the use of non-invasive, whole-brain data in an effort to reduce our reliance on invasively acquired data.

**Table 4:** A summary of studies using network models for epilepsy surgery.

**References**	**Network model**	**Patient number**	**Clinical data**	**Pathology**	**Findings**	**Comments**
Goodfellow et al. (29)	Wendling model	16	Ictal iEEG (grid) + numerical simulations	Various, lesional and nonlesional	Predict surgical outcome. Alternative or optimal surgical strategy can be offered	First attempt on clinical data in this series
Lopes et al. (32)	Wendling + Theta model	16	Peri-ictal & Ictal iEEG (grid) + numerical simulations	Various, lesional and nonlesional	Alternative or optimal strategy may be offered by removing rich-club structures	Subsequent work of Goodfellow et al. (29)
Lopes et al. (35)	Theta model	16	Peri-ictal iEEG (grid)	Various, lesional, and nonlesional	Predict surgical outcome using a metric derived from network model	Subsequent work of Lopes et al. (32)
Lopes et al. (132)	Theta model	16	iEEG (grid)	Various, lesional, and nonlesional	SOZ is not a good predictor of EZ for focal epilepsies with a multi-focal nature	Subsequent work of Lopes et al. (35)
Lopes et al. (122)	Theta model	15	Scalp EEG	Various, lesional and nonlesional	Lateralization of EZ	Non-invasive EEG source space
Jirsa et al. (124)	Epileptor model	24	iEEG + data from animal model	Various, lesional, and nonlesional	Reproduce seizure propagation in brain networks as observed by iEEG	Propose the model
Proix and Jirsa (125)	Epileptor model	18	Ictal sEEG	Various, lesional, and nonlesional	Predict the seizure propagation	First attempt to use clinical data
Jirsa et al. (36)	Epileptor model + structural brain network	1	Ictal sEEG + structural neuroimaging data	Nonlesional	Individualized model, predict subset of brain structure responsible for seizure generation	Subsequent work of Jirsa et al. (124)
Proix et al. (30)	Epileptor model + structural brain network	15	Ictal sEEG + structural neuroimaging data	Various, lesional, and nonlesional	Structural networks are able to explain change in functional connectivity	Subsequent work of Jirsa et al. (124)
Wendling et al. (129)	Wendling model	5	Ictal sEEG + numerical simulations	mTLE (lesional and nonlesional)	Theoretical model produces realistic epileptic signals that match sEEG recordings from mTLE	The original theoretical work along with data validation
Wendling et al. (136)	Wendling model + Functional connectivity	1	sEEG	mTLE	Potential to identify epileptogenic networks	Subsequent work of Wendling et al. (129)
Wendling et al. (139)	Wendling model	1	sEEG + animal model	mTLE	Replicate observed signals and predict the mechanisms validated by experiments and clinical data	A multi-level computational model

## 6. Discussion

Dynamical network models have the potential to improve characterization and delineation of the EZ. While initially based on iEEG recordings, these models have more recently been extended to the analysis of non-invasive EEG and MEG whole-brain recordings that, unlike iEEG, are not affected by limited spatial sampling, nor sensor positions.

### 6.1. Advantages of This Approach

Dynamical network modeling approaches represent an important shift away from a subjective interpretation of iEEG recordings toward an objective quantification of the putative EZ with their novel analyses of EEG and MEG interictal and ictal electrophysiological signals. By testing the effects of candidate epileptogenic nodes on network excitability and seizure transition states, these approaches permit deliberate, step-wise hypothesis testing of neural pathways that are critical for seizure generation and propagation before any surgical intervention takes place (29, 31, 32, 35). And, while not the focus of this review, in patients who are not deemed surgical candidates, these approaches may still be useful for neuromodulation targets. Recent work from Li et al. (40) and Scheid et al. (39) suggests “weak” nodes can be identified using network models for which neuromodulation strategies may be devised to reduce seizure susceptibility. Further study is required to clinically validate this concept. The interrogation of whole-brain structural and functional networks overcomes the major limitation of traditional invasive monitoring that is highly dependent on the implicit assumption that iEEG electrodes are placed in the ideal position for accurate delineation of the EZ (20). The approach also minimizes the influence of subjective clinical interpretation of seizure semiology in the pre-surgical work-up of these patients. For pre-operative planning, the quantifiable nature of dynamical network modeling facilitates an objective comparison with traditional non-invasive methods of EZ mapping, such as PET (positron emission tomography) and SPECT (single-photon emission computed tomography).

### 6.2. Limitations of This Approach

There are several limitations of dynamical network modeling combined with EEG and MEG source imaging. As discussed previously, field spread and signal leakage reduces the spatial resolution of source solutions and may limit the capacity of models to accurately identify the EZ (109, 140). Modeling is also dependent on the acquisition of high quality EEG or MEG interictal and ictal signals with minimal noise and artifact interference (141, 142). As also noted, all network models have underlying mathematical and physiological assumptions that may not be entirely valid such that, to date, no favored systematic approach exists (33). The veracity of these assumptions can only be rigorously tested with prospective epilepsy surgery studies, which are currently lacking. Indeed, dynamic network modeling is still in its infancy and the relationship between structural networks and functional networks is not yet clear, particularly with respect to a complex problem such as epilepsy. To date, these approaches cannot reliably distinguish between different anatomical structures based on the specific pathology.

### 6.3. Next Steps

Multi-modal neuroimaging techniques have assisted pre-surgical characterization of the putative EZ in pharmaco-refractory focal epilepsy. Better techniques are needed for the more challenging patients with MRI-normal and complex lesional focal epilepsy (141, 143, 144). To this end, network analysis and dynamical network models have shown considerable promise with their more objective computational approach to finding a surgical solution in these difficult cases (29–31). As pointed out here and by others (13, 18, 50, 113, 145), large cohorts are required to assess the effectiveness of these approaches in the clinical setting. Dynamical modeling may further assist by combining with different neuroimaging techniques, such as fMRI and tractography, to better model patient-specific brain structures and pathological dynamics to improve the efficacy and clinical utility of epilepsy surgery. How such a combined approach provides clinical value is yet to be fully elucidated but recent achievements by Jirsa et al. (124) and Proix et al. (30) demonstrate the merit of incorporating functional and structural information into the predictive model. It is conceivable that whole brain dynamic network modeling approaches may eventually render intracranial exploration unnecessary or even obsolete. The limitations of intracranial monitoring in its current forms disqualifies it as a true gold standard for mapping EZ networks. The evolution of more sophisticated whole-brain dynamic modeling approaches, which can overcome the sampling problem, might establish a new standard for pre-surgical epilepsy planning that is closer to the ground truth for unraveling EZ pathways. Potential benefits for epilepsy surgery patients might include reduced peri-operative morbidity and improved post-operative outcome. Routine clinical application might help elucidate the structural and functional substrates that link seizure semiology to seizure onset and propagation (146) with less clinical subjectivity to the point where elements of the semiology, not routinely included in existing models, could refine future network modeling strategies.

## 7. Conclusion

This review provides an update on the emerging roles of network analysis and dynamical network modeling in the surgical work-up of patients with pharmaco-resistant epilepsy. While still in their relative infancy, these novel approaches lend more objectivity to identification of the epileptogenic zone and they add much-needed specificity and flexibility to hypothesis testing of neural networks that are involved in epileptogenesis at the individual patient level in the spirit of twenty-first century “precision” medicine. The increasing sophistication of structural and functional connectivity analysis (from MRI, fMRI, DTI, EEG, and MEG) has paved the way for the evolution of many promising dynamical network modeling strategies. Most importantly, in the clinical context of epilepsy surgery, the aim is to improve patient evaluation and perform a successful resection that grants patients long-term seizure freedom for a better quality of life. The potential clinical impact of dynamical network modeling to improve post-surgical outcomes and to limit the subjectivity and invasiveness tied to current-day intracranial monitoring will only be realized with successful translation of these approaches to large prospective clinical studies.

## Author Contributions

MCa: writing original draft. MCa, SV, AP, WW, MCo, and CP: writing review and editing. All authors contributed to the article and approved the submitted version.

## Conflict of Interest

The authors declare that the research was conducted in the absence of any commercial or financial relationships that could be construed as a potential conflict of interest.

## Publisher's Note

All claims expressed in this article are solely those of the authors and do not necessarily represent those of their affiliated organizations, or those of the publisher, the editors and the reviewers. Any product that may be evaluated in this article, or claim that may be made by its manufacturer, is not guaranteed or endorsed by the publisher.
